# Activated ERM Protein Plays a Critical Role in Drug Resistance of MOLT4 Cells Induced by CCL25

**DOI:** 10.1371/journal.pone.0052384

**Published:** 2013-01-09

**Authors:** Li Zhang, Ruijing Xiao, Jie Xiong, Jun Leng, Altaf Ehtisham, Yi Hu, Qianshan Ding, Hui Xu, Shengwu Liu, Jin Wang, Dean G. Tang, Qiuping Zhang

**Affiliations:** 1 Department of Immunology, School of Basic Medical Science, Wuhan University, Wuhan, China; 2 Department of Molecular Carcinogenesis, The University of Texas M.D. Anderson Cancer Center, Smithville, Texas, United States of America; Shanghai Jiao Tong University School of Medicine, China

## Abstract

We have previously demonstrated that the CCR9/CCL25 signaling pathway plays an important role in drug resistance in human acute T-lymphocytic leukemia (T-ALL) by inducing activation of ERM protein with polarized distribution in T-ALL cell line MOLT4. However, the mechanism of action of the activated ERM protein in the drug resistance of MOLT4 cells induced by CCL25 remains uncharacterized. Here we investigated the mechanism of CCR9/CCL25-initiated drug resistance in CCR9-high-expressing T-ALL cells. Our results showed that 1) the function of P-gp was increased after treatment with CCL25; 2) P-gp colocalized and co-immunoprecipitated with p-ERM and F-actin in CCL25 treated cells; and 3) ERM-shRNA conferred drug sensitivity coincident with release of ERM interactions with P-gp and F-actin after treatment with CCL25. These data suggest it is pivotal that P-gp associate with the F-actin cytoskeleton through p-ERM in CCR9/CCL25 induced multidrug resistance of T-ALL cells. Strategies aimed at inhibiting P-gp-F-actin cytoskeleton association may be helpful in increasing the efficiency of therapies in T-ALL.

## Introduction

Acute T lymphoblastic leukemia (T-ALL) is a hematologic malignancy occurring mostly in children with poor prognosis [1], [2]. Resistance to anticancer drugs is an important cause of treatment failure in T-ALL. Therefore, circumventing drug resistance is likely to improve chemotherapy efficacy.

One of the major clinical obstacles in the treatment of hematologic malignancies is multidrug resistance (MDR). Classical MDR is the consequence of overexpression of transporter proteins, which belong to the family of ATP binding cassette (ABC) protein pumps and include P-glycoprotein (P-gp) and MDR related proteins. These proteins function to extrude the antitumor agents from the cytoplasm such that the multidrug resistant cells characteristically exhibit reduced levels of intracellular accumulation of drugs and show reduced cytotoxicity when compared with the parental cells [3]. Human P-gp, which is encoded by the MDR1 gene and represents a 170 kDa glycosylated integral plasma membrane protein, plays a major role in causing MDR in leukemia cells [4]. It is reported that the expression and polarized distribution of P-gp are involved in its extrusion function [5], [6].

Ezrin/radixin/moesin (ERM) protein family, which cross-links actin filaments with plasma membrane proteins, is involved in the organization of the cytoskeleton. ERM proteins are highly expressed and exhibit different intracellular localizations in various malignant tumors [7]. Interaction between plasma membrane molecules and cytoskeleton may play an essential role in membrane trafficking, signal transduction and various cellular functions, including cell motility and apoptosis [8]–[11]. Some evidence suggests that the actin-filament association with a variety of cellular proteins is mediated by ERM proteins [8]. There is also growing evidence that the cytoskeleton functions intimately in the P-gp-mediated MDR [12], [13]. Notably, the MDR pumps are usually localized at polarized sites of epithelial cells together with some membrane proteins [14]. The polarization of these proteins depends on the distribution of the cytoskeleton, as well as on the interaction of the pump-associated proteins with the actin cytoskeleton through ERM proteins [15].

CCR9, a member of G protein-coupled receptors, is crucial in T-cell development and for tissue-specific homing of T cells upon binding to its specific ligand CCL25 [16]. CCR9 is highly expressed in MOLT4 cells, a T-ALL cell line. Our previous studies showed that CCL25 can induce chemotherapeutic drug resistance in T-ALL [17] and that CCL25 can effectively induce polarization of MOLT4 cells with redistribution of ERM proteins after activation [7]. Herein, using the MOLT4 cells as a model, we investigate the potential interactions between P-gp and actin cytoskeleton through activated ERM proteins and the role of these interactions in CCR9/CCL25 mediated MDR in MOLT4 cells. Specifically, we aim to determine, in MOLT4 cells treated with CCL25, (a) the functions of P-gp; (b) the cellular distribution of and potential interactions among P-gp, ERM proteins, and F-actin; and (c) the effect of ERM-shRNA on susceptibility to drug-mediated cytotoxicity, drug efflux, and localization of P-gp, as well as the interaction with F-actin. Our results revealed that (1) the function of P-gp was enhanced after treatment with CCL25, (2) P-gp polarized, colocalized, and co-immunoprecipitated with p-ERM and F-actin in CCL25 treated cells, and (3) treatment with ERM-shRNA induced drug susceptibility, and P-gp molecule redistribution and dissociation from F-actin. These data suggest that P-gp association with the F-actin cytoskeleton through the activated ERM proteins is pivotal in CCR9/CCL25 induced MDR in MOLT4 T-ALL cells. Interference of the interactions between the P-gp and F-actin cytoskeleton may hold potential for T-ALL therapy.

## Materials and Methods

### Cell lines and cell culture

Human acute lymphoblastic leukaemia cell line MOLT4 (which naturally express high levels of CCR9) was obtained from ATCC and cultured in RPMI 1640 medium (Hyclone, USA) supplemented with 10% FBS (Gibco, USA), penicillin (100 U/ml) and streptomycin (100 µg/ml). The doxorubicin (DOX)-resistant MOLT4 cells (MR) were developed by exposing the parental MOLT4 cells, stepwise, to increasing concentrations of DOX according to the reported protocol [18]. Reselection of the resistant cell line was performed twice a week by exposure to 1 µg/ml DOX.

All cells were cultured in a humidified incubator at 37°C with 5% CO_2_. Only cells in the logarithmic phase of growth were used for experiments. The MR cells were maintained without DOX for at least 48 h before the experiments.

### Evaluation of P-gp functions

Accumulation and efflux experiments were conducted to assess the functions of P-gp. Briefly, cells (1×10^6^) were cultured in drug-free medium for 24 h prior to analysis. On the following day, cells were washed with PBS, incubated with 100 ng/ml CCL25 (R&D System) or anti-CCR9 Ab (Abcam, UK) and CCL25, and then treated with or without verapamil (VRP, Sigma, USA) for 1 h before treatment with 0. 5 µg/ml DOX or 2 µM Rhodamine123 (Rh123). In accumulation experiments, cells were washed twice in ice-cold PBS and analyzed by flow cytometry and/or laser scanning confocal microscopy (LSCM) immediately after the above-mentioned treatments. In the efflux experiments, cells were cultured in a drug-free medium for 1 h. The amount of remaining intracellular drug accumulation was detected by flow cytometry (FCM). This indirectly reflected the drug effluxing role of P-gp.

### RNA preparation and reverse transcriptase polymerase chain reaction (RT-PCR)

Total RNA was isolated from control or treated MOLT4 cells using TRIZOL Reagent (Invitrogen, Merelbeke, Belgium) according to the manufacturer's instructions. The concentration and purity of total RNA were determined using spectrophotometry. RT was carried out with 500 ng of total RNA from each sample using the RNA PCR Kit (TaKaRa Biotechnology Co., Ltd.). The investigated mRNAs included those coding for P-gp, ezrin, radixin, moesin, and GAPDH (primer information and PCR conditions are listed in Table 1). PCR products (10 µl) were separated on 3% agarose gels, and the gel imaging system (Vilber Lourma, France) was used to scan the gel and quantify the levels of expression. Amplification of GAPDH was used as the control.

**Table 1:** Primer sequences and RT-PCR conditions.

Genes	Primer	Conditions
P-gp	Sense: 5′-CCCATCATTGCAATAGCAGG-3′	94°C for 30 s, 50°C for 30 s and 72°C for 30 s (35 cycles)
	Antisense: 5′-GTTCAAACTTCTGCTCCTGA-3′	
Ezrin	Sense:5′-GAATACACTGCCAAGATTGC-3′	94°C for 30 s, 56°C for 45 s and 72°C for 45 s (35 cycles)
	Antisense: 5′-CTCATGTTCTCGTTGTGGAT-3′	
Radixin	Sense: 5′-GCTAGGTGTTGATGCTTTGG-3′	94°C for 30 s, 52°C for 30 s and 72°C for 40 s (35 cycles)
	Antisense: 5′-GACGTTCCATTAGCTCTTCC-3′	
Moesin	Sense: 5′- AGCCTTGGCATCTAGAGCTTGATGC -3′	94°C for 45 s,58°C for 45 s and 72°C for 1 min (35 cycles)
	Antisense: 5′- GGACTGAACCCTGGGGAGAAGACA-3′	
GAPDH	Sense: 5′-CCATGGAGAAGGCTGGGG-3′	94°C for 30 s, 65°C for 30 s and 72°C for 40 s (35 cycles)
	Antisense: 5′-CAAAGTTGTCATGGATGACC-3′	

### SDS-PAGE and Western Blot Analysis

MOLT4 cells were treated with 100 ng/ml of CCL25 for 10 min. MR cells and untreated MOLT4 cells were used as controls. Whole cell lysate was prepared by lysing the cells in the modified RIPA (radioimmunoprecipitation assay) buffer (0.1% SDS, 1% sodium deoxycholate, 1% NP-40, 150 mM NaCl, 50 mM Tris-HCl pH 7.5 and a cocktail of protease inhibitors) followed by centrifugation at 12,000 *g* for 15 min. Subsequently, 40 µg of total proteins in the supernatant was separated by 10% SDS polyacrylamide gel electrophoresis after protein concentrations were determined by BCA protein assay kit (Beyotime, China). Separated proteins were transferred to Polyvinylidene Fluoride membranes (Schleicher and Schuell, Dassel, Germany), which were incubated in blocking buffer (5% nonfat milk dissolved in Tris-buffered saline and 0.1% Tween-20) for 1 h at room temperature. After blocking, membranes were incubated with primary Abs against P-gp, or beta-actin overnight at 4°C. Then, membranes were washed with TBST buffer (0.1% Tween-20 in TBS buffer) for six times. After washing, membranes were incubated with HRP-conjugated goat anti-mouse IgG Ab (1∶5000) for 1 h at room temperature. Following incubation, membranes were washed six times with TBST buffer. Signals were detected using enhanced chemiluminescence detection reagents following the manufacturer's instructions. Band intensity was measured by densitometry using the Quantity One software (Bio-Rad, Hercules, CA) by normalizing to the corresponding beta-actin levels. The western blot analysis was repeated twice.

### Flow cytometry (FCM)

MOLT4 cells were pretreated with or without 100 ng/ml of CCL25 for 10 min. MR cells were used as the positive control. Cells were then incubated with PE-labelled anti-P-gp Ab (eBioscience, USA) at 5 µg/ml in PBS for 30 min at 4°C, followed by washing in ice-cold PBS twice and finally resuspended in 500 µl PBS for flow cytometric analysis (Beckman, USA). As control, cells were stained with the matching isotype control Abs.

### Laser scanning confocal microscopy (LSCM)

To observe the co-localization of P-gp, phospho-ERM (p-ERM; i.e., activated ERM) and F-actin, cells were pretreated with or without 100 ng/ml CCL25 or both CCL25 (100 ng/ml) and 1 µg/ml anti-CCR9 Ab (Abcam, UK). Cells were plated and cultured on polylysine-coated coverslips. To label P-gp protein, cells were first fixed with 10% neutral formalin in PBS for 10 min at room temperature followed by incubation with an anti-P-gp Ab at 4°C. To observe the intracellular p-ERM, we performed a trichloroacetic acid fixation method [19] in order to inactivate phosphatases and maintain adequate levels of p-ERM proteins during sample processing. Briefly, cells were fixed in 10% trichloroacetic acid solution for 15 min at room temperature, and washed three times with PBS. After washing, cells were permeabilized with 0.2% Triton-X 100/PBS followed by incubation with anti-p-ERM or anti-F-actin Abs overnight at 4°C. After washing in PBS, samples were incubated with fluorescein-linked secondary Abs for 45 min at room temperature and examined under a confocal microscope (Leica, Germany).

### Co-immunoprecipitation analysis

Cells were first lysed in RIPA buffer for 1 h at 4°C under rotating conditions. After insoluble materials were removed by centrifugation at 12,000 g for 15 min, the soluble supernatants were precleared with protein A+G agarose beads to prevent non-specific binding. After 1-h incubation at 4°C, samples were centrifuged at 2,500 rpm to pellet the agarose beads, and the supernatants were saved for immunoprecipitation. After determining the protein concentration by BCA protein assay kit, 500 µg of total proteins were incubated with 1 µg of primary Abs (to P-gp, p-ERM, F-actin) and 100 µl protein A+G agarose beads overnight at 4°C under rotating conditions. The immunoprecipiate was collected by centrifugation at 2,500 rpm for 15 min at 4°C, washed in RIPA buffer, and finally resuspended in 40 µl of SDS-PAGE sample loading buffer. The immunoprecipitated proteins were detected by western blotting analysis.

### Plasmid construction and cell transfection

Short-hairpin RNAs (shRNAs, Table 2) to ezrin, radixin and moesin genes were constructed by Shanghai Genechem Company, which were cloned into pGCsilencer™ H1/Neo/RNAi vector. For transfection, 4×10^5^ cells were plated in 24-well plates without antibiotics. Transfections were performed with Lipofectamine 2000 (Invitrogen) according to the manufacturer's instructions. Media was replaced with OPTI-MEM1 (Invitrogen) supplemented with 1% (vol/vol) FBS 4–6 h after transfection. After 24 h, the medium containing 200 µg/ml G418 was applied for effective stable selection. In some experiments, several plasmids were co-transfected into cells and the stably transfected cell lines were established after 8 weeks of G418 selection. RT-PCR and western blotting assays were employed to analyze ezrin, radixin and moesin mRNA and protein levels, respectively.

**Table 2:** The information of shRNAs to ezrin,radixin,moesin genes.

Name	Stem	Loop	Stem
Ezrin	CCTGGAAATGTATGGAATCAA	CTCGAG	TTGATTCCATACATTTCCAGG
Radixin-Moesin	GCAGACAATTAAAGCTCAGAA	CTCGAG	TTCTGAGCTTTAATTGTCTGC

### Cell viability assays

We plated 2×10^3^ stable shRNA transfected MOLT4 or control vector transfected MOLT4 cells into 96-well plates. Cells were mixed with different concentrations of DOX and incubated for 48 h, after which 10 µl WST-1 reagents were added to each well and incubated at 37°C for 4 h. At the end, the absorbance was measured using a PerkinElmer 2030 VICTOR X Multilabel Plate Reader at 450 nm.

### Apoptosis assays

Apoptosis was determined using the Annexin-VFLUOS Staining Kit (Roche) allowing quantification by flow cytometry. In brief, ERM-shRNA or control vector transfected MOLT4 cells (5×10^5^ in 2 ml) grown in 6-well plates were treated with 1 µg/ml Vincristine (VCR) for 48 h. After washing, cells were incubated in 500 µl of binding buffer containing 10 µl of Annexin V-FITC and 5 µl of PI for 15 min at 37°C in the dark according to the manufacturer's instructions. Apoptosis was immediately quantified using FCM. Results were presented as percentages of Annexin V-FITC positive cells, or Annexin V-FITC and PI double positive cells.

### Statistical analysis

All experiments were repeated at least three times. Differences between 2 treatment groups were analyzed by Student's *t*-test. Data were expressed as mean ± S.D and P<0.05 was considered statistically significant.

## Results

### Drug resistance in MOLT4 cells was enhanced by CCL25/CCR9

**Figure 1:**
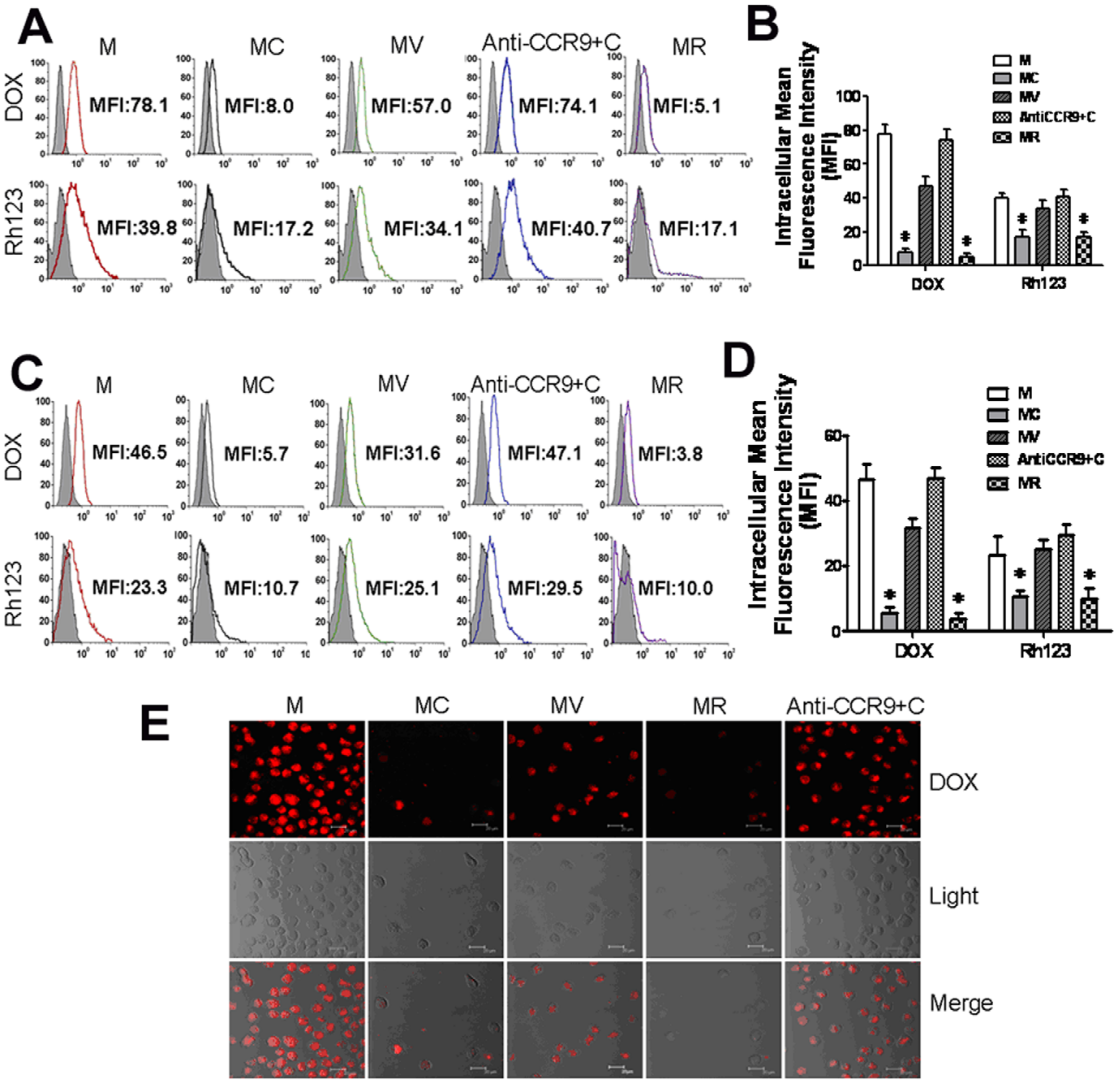
P-gp functions in MOLT4 cells treated with different factors. (A) Intracellular drug accumulation in untreated MOLT4 cells or MOLT4 cells treated with CCL25/verapamil/anti-CCR9 by FCM. These cells were incubated with DOX or Rh123, washed and the MFI determined by flow cytometry using MR cell line as a control. (B) The accumulation of DOX and Rh123 in MOLT4 cells. Values are mean ± SD. *, P<0.05. (C) Efflux assessment of DOX and Rh123 in MOLT4 cells detected by FCM. Cells were incubated with DOX or Rh123 as described above followed by washing. After 1 h of dye efflux at 37°C in the medium without DOX or Rh123, cells were washed twice with cold PBS and immediately analyzed using the same procedure as for the accumulation experiments. (D) The efflux of DOX and Rho123 in MOLT4 cells. Values are mean ± SD. *, p<0.05. (E) Intracellular accumulations in MOLT4 cells detected by LSCM. Cells were washed with PBS, incubated with or without verapamil before treatment with DOX, and then the cells were treated with CCL25 or anti-CCR9 antibody and CCL25. Confocal images (top); Nomarski differential interference contrast images (middle); and overlays of confocal and differential interference contrast images (bottom). Bar = 20 µm. All the results presented are the means of three independent experiments. M, MOLT4; MC, MOLT4 cells treated with CCL25; MV, MOLT4 cells treated with CCL25 and VRP; MR, MOLT4 cells treated with DOX for 9 months.

We previously reported that CCL25 selectively enhanced the resistance of MOLT4 and other T-ALL cells to TNF-α-mediated apoptosis [17]. To determine whether activation of CCR9, the receptor that binds CCL25, was involved in drug resistance in MOLT4 cells, we first performed accumulation and efflux assays to assess the function of P-gp. As shown in Fig. 1A, MOLT4 cells treated with CCL25 significantly decreased the drug accumulation. In untreated MOLT4 cells, the mean fluorescence intensity (MFI) for DOX and Rh123 was high and reached to 78.1 and 39.8, respectively. In contrast, in MOLT4 cells treated with CCL25, the MFI for DOX and Rh123 accumulation was 8.0 and 17.2, respectively (Fig. 1A). When cells were pretreated with an anti-CCR9 Ab followed by CCL25 stimulation, drug accumulation was maintained at high levels (Fig. 1A and B). Similar results were obtained when analyzed using LSCM (Fig. 1E). These results indicated that CCR9/CCL25 signaling reduces drug accumulation in MOLT4 cells.

Next, we performed drug efflux assays to measure the exclusion of DOX and fluorescent agent Rh123 from the cells to assess the functional activity of P-gp. We found that the drug-resistant MOLT4 cells again accumulated less DOX and Rh123 (Fig. 1C and D), suggesting that the P-gp was functional in our cell systems. In addition, when the cells were pretreated with P-gp inhibitor verapamil for 1 h [20], the efflux was obviously inhibited (data not shown), which underlines the importance of P-gp in the drug resistance of MOLT4 cells induced by CCR9/CCL25.

### Drug resistance induced by CCL25 is not accompanied by altered expression of P-gp

**Figure 2:**
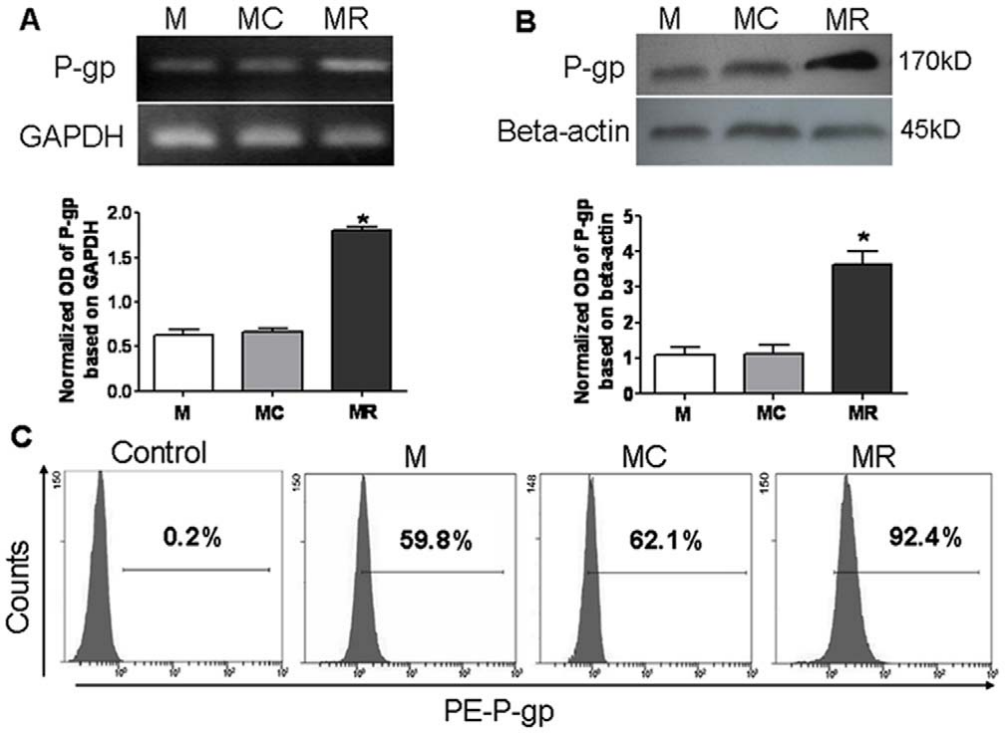
P-gp expression induced by CCL25 in MOLT4 cells. (A) RT-PCR and (B) Western blot analyses for P-gp expression induced by CCL25 in MOLT4 cells. MR cells were used as positive control. Amplification of GAPDH was used as control for RT-PCR assays. For loading control in western blot, membranes were incubated with monoclonal anti-β-actin antibody. (C). Expression of P-gp in cells stained with PE-anti-human MDR1 antibody by FCM. Values presented are the means of four independent experiments.

We employed RT-PCR, Western blot and FCM to determine whether CCL25-induced drug resistance might be caused by changes in expression of the P-gp. Surprisingly, there was no change in P-gp expression in MOLT4 cells treated with CCL25 although we detected high levels of its expression in the positive control MR cells (Fig. 2A–C). This result suggested that altered expression levels of P-gp may not be involved in the CCL25-induced drug resistance in MOLT4 cells.

### P-gp, p-ERM and F-actin colocalized and interacted with one another in CCL25-stimulated MOLT4 cells

**Figure 3:**
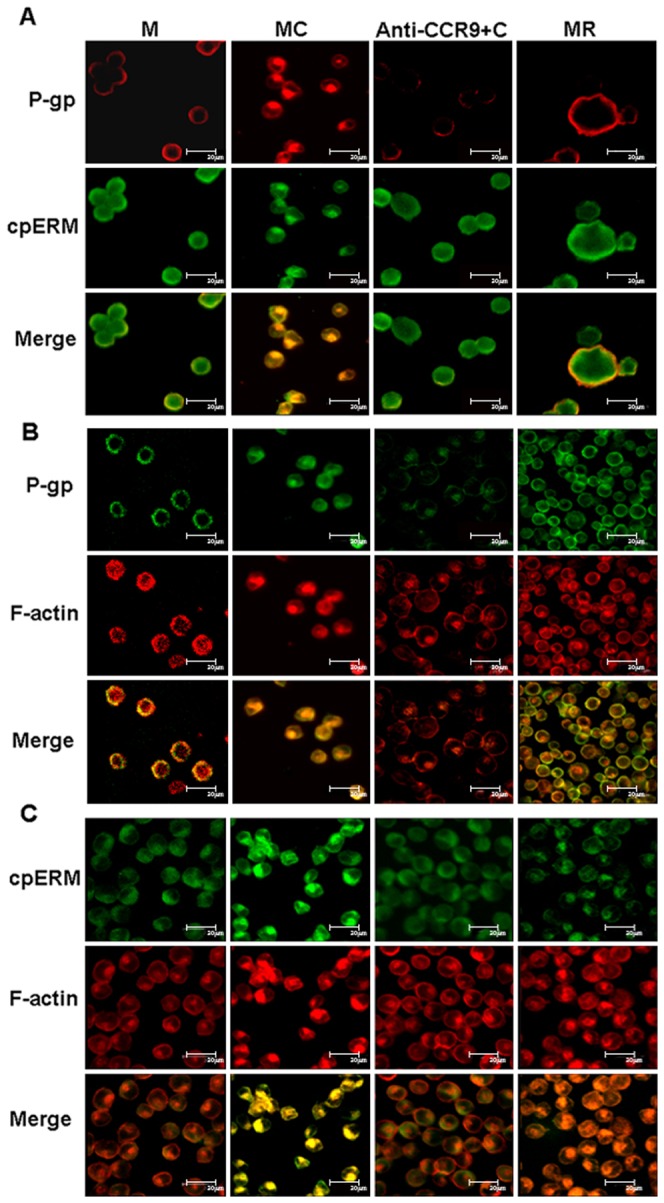
Colocalization of P-gp, p-ERM and F-actin in MOLT4 cells treated with CCL25 by confocal. Shown are the subcellular localizations of (A) P-gp (red) and p-ERM (green), (B) P-gp (green) and F-actin (red), and (C) p-ERM (green) and F-actin (red) in MOLT4 cells treated with CCL25 or anti-CCR9 and CCL25 together. All the images showed that P-gp and p-ERM or F-actin colocalized in CCL25 treated cells, and this colocalization could be abolished by anti-CCR9 antibody. Bar = 20 µm. The results represented the means of four independent experiments.

Subsequently, we investigated the subcellular distribution of P-gp by LSCM in MOLT4 cells treated with or without CCL25. In untreated cells, P-gp was mostly found homogeneously on the plasma membrane of MOLT4 cells whereas the p-ERM and F-actin were detected in the cytoplasm (Fig. 3A). In sharp contrast, MOLT4 cells stimulated with CCL25 expressed the P-gp selectively at the protruding or polarized part of MOLT4 cells (Fig. 3A and B), where p-ERM and F-actin were also colocalized (Fig. 3C). When the cells were pretreated with an anti-CCR9 Ab prior to CCL25 stimulation, the co-distribution patterns of P-gp, p-ERM and F-actin were disrupted. These results suggested that CCL25-induced drug resistance in MOLT4 cells may involve a redistribution of P-gp on the plasma membrane and colocalization of P-gp with p-ERM protein and F-actin.

**Figure 4:**
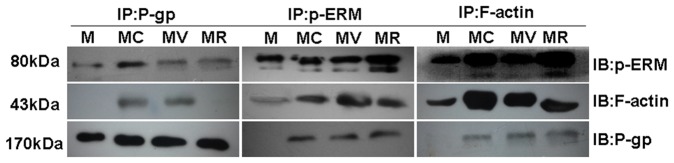
P-gp, ERM and F-actin co-immunoprecipitation induced by CCL25 in MOLT4 cells. Western blot for p-ERM, F-actin, and P-gp in P-gp (left panel), p-ERM (middle panel) and F-actin (right panel) immunoprecipitations, respectively, from lysates of MOLT4 cells in the presence or absence of CCL25.

We carried out co-immunoprecipitation experiments to further assess the level of P-gp association with p-ERM and F-actin molecules. A monoclonal Ab against F-actin detected an expected ∼43-kD band in the immunoprecipitates of P-gp in CCL25 treated cell lysate but not in untreated control lysate (Fig. 4, left). In addition, p-ERM was also detected in the immunoprecipitates of P-gp from CCL25 treated cell lysate with only very low amount of p-ERM detected in untreated cell lysate (Fig. 4, left). These results were confirmed by reciprocal co-immunoprecipitation experiments in which P-gp was detected in both F-actin and p-ERM immunoprecipitates from the CCL25 treated cell lysate (Fig. 4; middle and right panels). Our findings suggested that P-gp could be associated with the actin cytoskeleton and that this interaction may play an important role in drug resistance induced by CCL25.

### Silencing ERM proteins enhanced drug sensitivity and apoptosis of MOLT4 cells

**Figure 5:**
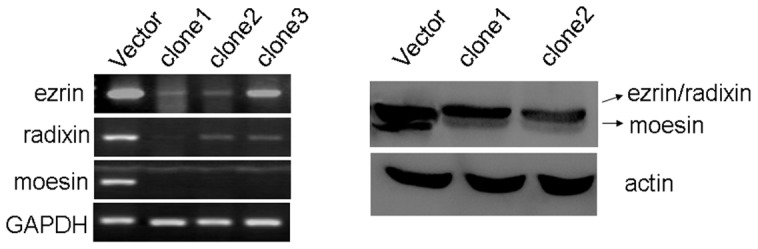
Effects of ERM-shRNA on ERM expression. MOLT4 cells were transfected with ERM-shRNAs and cells were selected in G418 (200 µg/ml) for 2 weeks. After 6–8 weeks with G418 selection, the clones were selected for identification by RT-PCR and Western blot.

To address the causal role of ERM proteins in chemokine-mediated drug resistance, we established stable MOLT4 cell clones in which these molecules were silenced by specific shRNAs (Fig. 5). There was a significant reduction of ezrin, radixin, and moesin RNAs and proteins in both clone 1 and clone 2 compared with MOLT4 cells transfected with empty vector (Fig. 5). We therefore chose clone 2 for all subsequent experiments.

**Figure 6:**
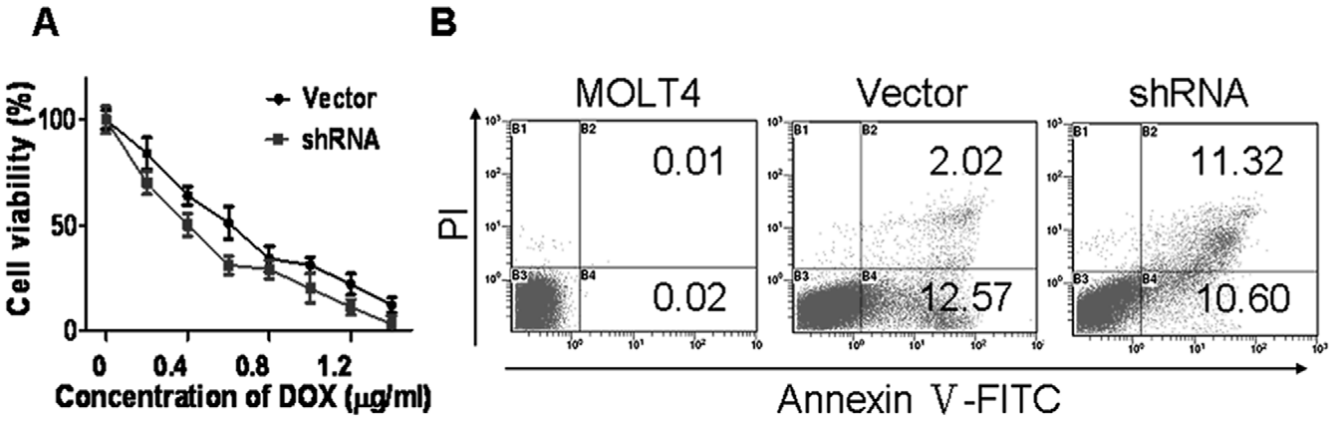
Cell viability and apoptosis analysis in ERM-silenced MOLT4 cells. (A) Cell viability was lower in ERM-silenced cells compared to empty vector-transfected cells. (B) The apoptosis rate in shRNA transfected cells was increased compared with empty vector-transfected cells.

To determine whether the biological characteristics have changed after transfection with shRNAs in MOLT4 cells, cell viability and apoptosis were evaluated by WST-1 and FCM. After the cells were incubated with different concentrations of DOX for 72 h, as shown in Fig. 6A, the viability of ERM-silenced cells was lower than that of empty vector-transfected cells (P<0.05), indicating that ERM silencing enhanced sensitivity of MOLT4 cells to DOX treatment. Moreover, when cells were treated with VRP for 24 h, we observed increased apoptosis in shRNA transfected cells compared with the vector-transfected cells (Fig. 6 B).

### ERM proteins participated in CCL25-induced drug resistance in MOLT4 cells

**Figure 7:**
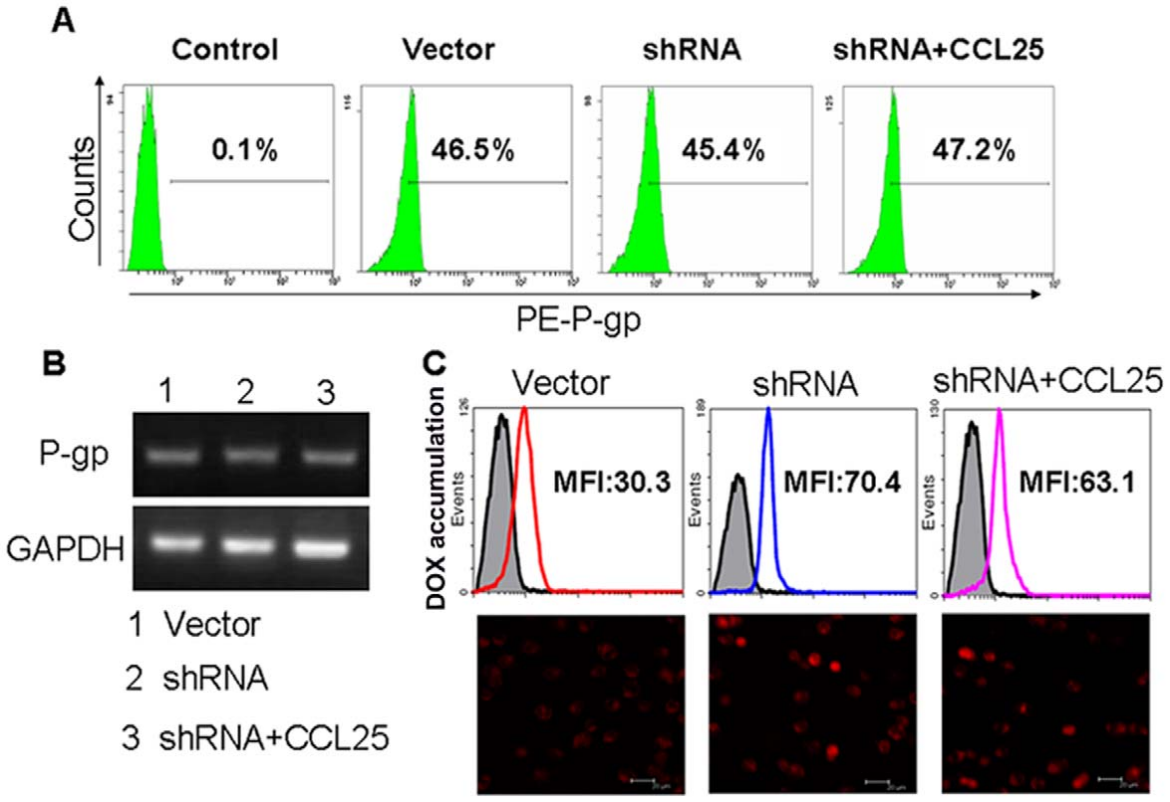
Expression and functionality of P-gp in ERM-silenced MOLT4 cells treated with CCL25. (A) Expression of P-gp by FCM. (B) Expression of P-gp by RT-PCR. (C) DOX accumulation by FCM (upper) and LSCM (bottom). Bar = 20 µm.

To ensure that the shRNAs to ERM proteins did not alter the expression of P-gp, we detected its expression by FCM and RT-PCR assays. As expected, the expression levels of P-gp in shRNA transfected cells did not change compared to empty vector-transfected cells, nor did the expression levels of P-gp also before and after CCL25 treatment (Fig. 7A–B). Next, we investigated drug accumulation in shRNA transfected cells, and found that the ERM-silenced MOLT4 cells showed dramatically increased drug accumulation as compared with empty vector-transfected control (Fig. 7C), which was corroborated by LSCM analysis (Fig. 7C). The results suggested that interference of ERM expression could increase drug accumulation, enhance apoptosis rate and reverse drug resistance.

**Figure 8:**
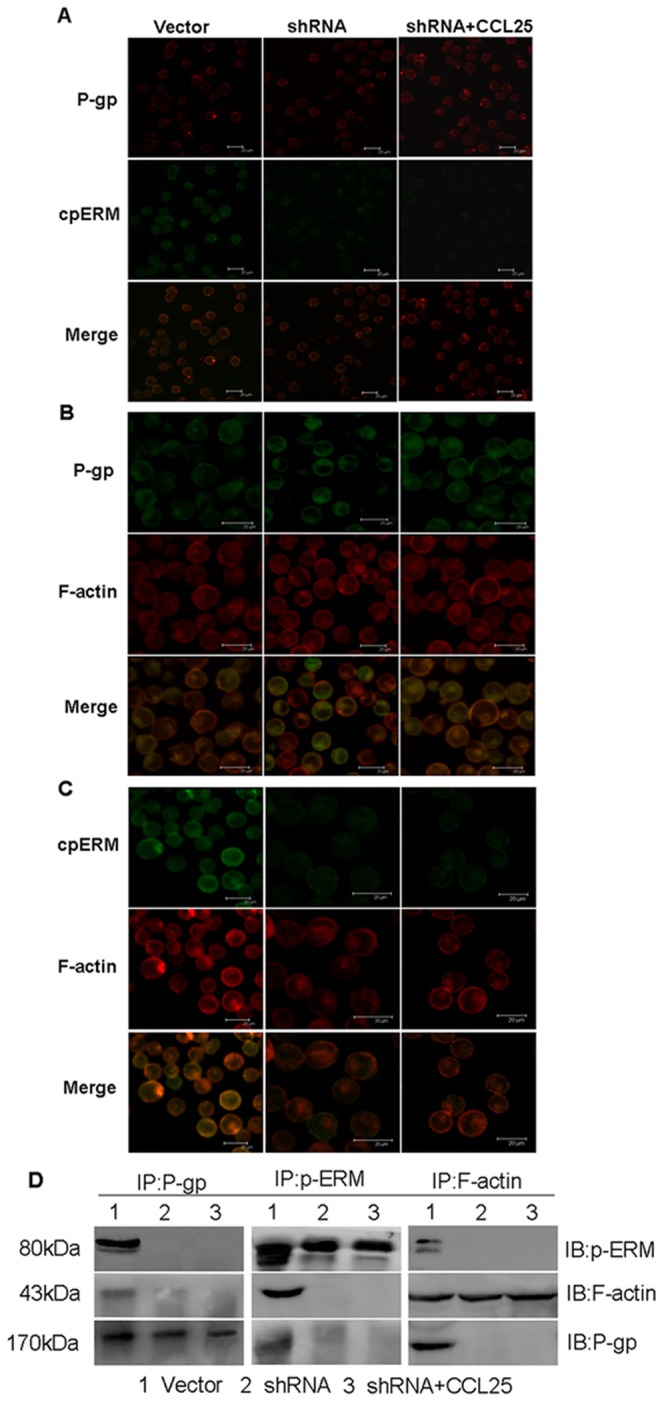
Role of P-gp-p-ERM-F-actin association in ERM-silenced MOLT4 cells treated by CCL25. Shown are the subcellular localizations of (A) P-gp (red) and p-ERM (green), (B) P-gp (green) and F-actin (red), (C) p-ERM (green) and F-actin (red) in ERM-silenced MOLT4 cells treated by CCL25. All images showed P-gp and p-ERM or F-actin distribution on the cells. Bar = 20 µm. (D) Co-immunoprecipitation analysis of ERM-silenced MOLT4 cells, either untreated or pretreated with CCL25.

Interestingly, when ERM-silenced MOLT4 cells were stimulated by CCL25, the intracellular accumulation of DOX did not reduce significantly, which was in contrast to the vector control cells treated with CCL25 (Fig. 7C). These results indicated that cytoskeletal regulatory proteins ERM might be involved in CCL25-induced drug resistance in MOLT4 cells. Consequently, we investigated the distribution of P-gp in ERM-silenced MOLT4 cells by LSCM. The results revealed that P-gp staining appeared unpolarized and localized all around the ERM-silenced cells, despite that the cells were stimulated with CCL25 (Fig. 8A–B). Furthermore, colocalization between p-ERM, F-actin and P-gp was no longer detectable in ERM-silenced MOLT4 cells treated by CCL25 (Fig. 8A–C). These data were consistent with our co-immunoprecipitation experiments and, in fact, after CCL25 treatment, P-gp was no longer detectable in both p-ERM and F-actin immunoprecipitates (Fig. 8D). These results strongly suggested that interactions between P-gp and cytoskeleton were involved in CCL25-induced drug resistance and activated ERM protein played an important role in this process.

## Discussion

In this study, using T-ALL cell line MOLT4 cells, we provided experimental evidence that P-gp association with the F-actin cytoskeleton through p-ERM is involved in CCL25/CCR9 induced MDR. Our findings suggested that ERM proteins were crucial components of drug resistance in leukemic cells induced by chemokines. Moreover, we found no relationship between P-gp expression levels and drug resistance but rather a functional link between P-gp and F-actin cytoskeleton during CCL25-induced drug resistance.

Resistance to cytotoxic drugs is a major obstacle in the treatment of hematologic malignancies [21], [22]. Mechanisms underlying MDR include, among others, enhancement of drug efflux carried out by membrane proteins (e.g., P-gp, MRP, and LRP) [23], [24], reinforcement in enzymatic detoxification of cytotoxic drugs by glutathione-s-transferase system [25]–[27], and inhibition of cell apoptosis [28], [29]. The ‘classic’ MDR is mediated by P-gp [4], [30], [31]. To date, little attention has been payed towards the relationship between chemokines and MDR at the cellular level in hematologic malignancies.

In the present study, we first noticed that the intracellular accumulation of DOX and Rh123 was significantly decreased following CCL25 treatment and this effect could be blocked by an anti-CCR9 Ab, which indicated that there was indeed a certain relationship between CCL25 and MDR in MOLT4 cells. Our findings provided strong evidence for developing CCR9 antagonists to overcome chemokine-induced MDR in leukemia cells. In addition, the results of efflux assays revealed that P-gp was functional and the inhibition by VRP substantiated that P-gp played a critical role in the drug resistance of MOLT4 cells induced by CCR9/CCL25. Overall, our data were in line with the prevailing view that P-gp functions intimately in MDR in cancer cells [4], [30], [31].

To our surprise, P-gp showed no alterations in the expression level but re-localized to the polarized plasma membranes on MOLT4 cells treated with CCL25. It had been reported that a polarized distribution of P-gp depended upon the rearrangement of cytoskeletal proteins such as actin and ERM [12], [13], [32]. Also, Vicky Goler-Baron and Assaraf had shown that the ERM protein complex selectively localized to the border of extracellular vesicle membranes, implicating a key role for the tethering of MDR pumps to the actin cytoskeleton [33]. We had previously shown that CCL25 effectively induced polarization of MOLT4 cells following activation of ERM proteins with polarized redistribution [7]. Since there was evidence that the redistribution of P-gp in association with ERM proteins played a role in MDR [12], we speculated that activated ERM proteins might be involved in the polarized distribution of P-gp during CCL25 stimulation.

Two pieces of evidence supported this speculation. First, the results of LSCM analysis indicated that P-gp, p-ERM and F-actin all co-localized at the polarized sites of CCL25-treated MOLT4 cells. Second, co-immunoprecipitation experiments confirmed direct interactions among these proteins. These findings were fully consistent with others' data independently showing colocalizations of ERM proteins, actin, and P-gp on the plasma membrane [34]–[36]. Our observations in turn suggested that P-gp-p-ERM-F-actin interactions played a pivotal role in regulating the localization of P-gp at well-defined membrane sites. Consistent with this suggestion, shRNA-mediated ERM silencing in MOLT4 cells significantly increased cell apoptosis. Intracellular accumulation of drugs was further increased in ERM-silenced MOLT4 cells treated with CCL25. Notably, there was no membrane polarization in ERM-silenced cells after treatment with CCL25, consistent with our previous work (7). Moreover, only a weak fluorescence of p-ERM was detected in ERM-silenced MOLT4 cells (Fig. 8, A and C), further confirming the role of ERM proteins. Finally, ERM silencing appeared to sever the P-gp linkage with F-actin, based on the results of LSCM and immunoprecipitation, further suggesting that p-ERM proteins played a critical role in P-gp-F-actin association in CCR9/CCL25-mediated drug resistance of MOLT4 cells. Taken together, these results suggested that 1) the activated state of ERM was key for P-gp-mediated function, particularly in enhancing lymphoblastic leukemia cell sensitivity to drug treatment, and 2) the association of P-gp with F-actin through the p-ERM proteins was crucial for the maintenance of P-gp polarization in MDR induced by CCR9/CCL25 signaling pathway.

In this study, we observed that the P-gp expression level was significantly increased in long-term (9 months) DOX treatment cells (MR) even without CCL25 treatment. These observations are consistant with those made by Villar et al, which also demonstrated that DOX induced MDR is closely related with high levels of P-gp expression [37]. These findings, together, suggest that there may exist different mechanisms between short-term chemokine induced drug resistance vs. long-term DOX induced drug tolerance.

New therapeutic agents need to be developed to overcome MDR in the chemotherapy of leukemia. A growing number of methods are being developed to overcome intrinsic MDR of tumor cells. Protocols for overcoming tumor resistance mediated by chemokines are still at an early stage and require further research. Our results suggest that interfering with the interactions between P-gp and F-actin may represent a potentially novel way for overcoming chemokine-mediated MDR in T-ALL cells.

## References

[1] Pui CH, Behm FG, Crist WM (1993) Clinical and biologic relevance of immunologic marker studies in childhood acute lymphoblastic leukemia. Blood 82: 343–362. 8329694

[2] Willemse MJ, Seriu T, Hettinger K, d'Aniello E, Hop WC, et al (2002) Detection of minimal residual disease identifies differences in treatment response between T-ALL and precursor B-ALL. Blood 99 (12) 4386–4393. 12036866 10.1182/blood.v99.12.4386

[3] Gottesman MM, Ling V (2006) The molecular basis of multidrug resistance in cancer: the early years of P-glycoprotein research. FEBS Lett 580: 998–1009. 16405967 10.1016/j.febslet.2005.12.060

[4] Sharon FJ (2008) ABC multidrug transporters: structure, function and role in chemoresistance. Pharmacogenomics 9 (1) 105–127. 18154452 10.2217/14622416.9.1.105

[5] Meschini S, Calcabrini A, Monti E, Del Bufalo D, Stringaro A, et al (2000) Intracellular P-glycoprotein expression is associated with the intrinsic multidrug resistance phenotype in human colon adenocarcinoma cells. Int J Cancer 87 (5) 615–628. 10925353

[6] Arora A, Seth K, Shukla Y (2004) Reversal of P-glycoprotein-mediated multidrug resistance by dially1 sulfide in K562 leukemic cells and in mouse liver. Carcinogenesis 25 (6) 941–949. 14729595 10.1093/carcin/bgh060

[7] Zhou B, Leng J, Hu M, Zhang L, Wang Z, et al (2010) Ezrin is a key molecule in the metastasis of MOLT4 cells induced by CCL25/CCR9. Leukemia research 34 (6) 769–776. 20036004 10.1016/j.leukres.2009.11.025

[8] Bretscher A (1999) Regulation of cortical structure by the ezrin-radixin-moesin protein family. Curr Opin Cell Biol 11 (1) 109–116. 10047517 10.1016/s0955-0674(99)80013-1

[9] Saarikangas J, Zhao H, Lappalainen P (2010) Regulation of the actin cytoskeleton- plasma membrane interplay by phosphoinositides. Physiol Rev 90 (1) 259–289. 20086078 10.1152/physrev.00036.2009

[10] Luna EJ, Hitt AL (1992) Cytoskeleton-plasma membrane interactions. Science 258: 955–963. 1439807 10.1126/science.1439807

[11] Hébert M, Potin S, Sebbagh M, Bertoglio J, Bréard J, et al (2008) Rho-ROCK-dependent ezrin-radixin-moesin phosphorylation regulates Fas-mediated apoptosis in Jurkat cells. J Immunol 181 (9) 5963–5973. 18941185 10.4049/jimmunol.181.9.5963

[12] Luciani F, Molinari A, Lozupone F, Calcabrini A, Lugini L, et al (2002) P-glycoprotein-actin association through ERM family proteins: a role in P-glycoprotein function in human cells of lymphoid origin. Blood 99 (2) 641–648. 11781249 10.1182/blood.v99.2.641

[13] Brambilla D, Zamboni S, Federici C, Lugini L, Lozupone F, et al (2012) P-glycoprotein binds to ezrin at amino acid residues 149–242 in the FERM domain and plays a key role in the multidrug resistance of human osteosarcoma. Int J Cancer 130 (12) 2824–2834. 21780101 10.1002/ijc.26285

[14] Rodriguez-Boulan E, Nelson WJ (1989) Morphogenesis of the polarized epithelial cell phenotype. Science 245 (4919) 718–725. 2672330 10.1126/science.2672330

[15] Padányi R, Xiong Y, Antalffy G, Lór K, Pászty K, et al (2010) Apical scaffolding protein NHERF2 modulates the localization of alternatively spliced plasma membrane Ca2+ pump 2B variants in polarized epithelial cells. J Biol Chem 285 (41) 31704–31712. 20663896 10.1074/jbc.M110.164137 PMC2951242

[16] Tong X, Zhang L, Zhang L, Hu M, Leng J, et al (2009) The Mechanism of Chemokine Receptor 9 Internalization Triggered by Interleukin 2 and Interleukin 4. Cellular & Molecular Immunology 6 (3) 181–189. 19567201 10.1038/cmi.2009.25 PMC4003061

[17] Qiuping Z, Jei X, Youxin J, Wei J, Chun L, et al (2004) CC Chemokine Ligand 25 Enhances Resistance to Apoptosis in CD4+ T Cell from Patients with T-cell Lineage Acute and Chronic Lymphocytic Leukemia by means of Livin Activation. Cancer Research 64: 7579–7587. 15492285 10.1158/0008-5472.CAN-04-0641

[18] Liu ZL, Onda K, Tanaka S, Toma T, Hirano T, et al (2002) Induction of multidrug resistance in MOLT-4 cells by anticancer agents is closely related to increased expression of functional P-glycoprotein and MDR1 mRNA. Cancer Chemother Pharmacol 49 (5) 391–397. 11976833 10.1007/s00280-001-0411-5

[19] Hayashi K, Yonemura S, Matsui T, Tsukita S (1999) Immunofluorescence detection of ezrin/radixin/moesin (ERM) proteins with their carboxyl-terminal threonine phosphorylated in cultured cells and tissues. J Cell Sci 112 (Pt 8) 1149–1158. 10085250 10.1242/jcs.112.8.1149

[20] Tsuruo T, Iida H, Tsukagoshi S, Sakurai Y (1981) Overcoming of vincristine resistance in P388 leukemia in vivo and in vitro through enhanced cytotoxicity of vincristine and vinblastine by verapamil. Cancer Res 41 (5) 1967–1972. 7214365

[21] Galski H, sivan H, Lazarovici P, Nagler A (2006) In vitro and in vivo reversal of MDR1-mediated multidrug resistance by KT-5720: Implications on hematologic malignancies. Leukemia Research 30 (9) 1151–1158. 16542724 10.1016/j.leukres.2006.02.016

[22] Asakura K, Uchida H, Miyachi H, Kobayashi H, Miyakawa Y, et al (2004) TEL/AML1 overcomes drug resistance through transcriptional repression of multidrug resistance-gene expression. Molecular Cancer Research 2 (6) 339–347. 15235109

[23] Gottesman MM, Fojo T, Bates SE (2002) Multidrug resistance in cancer: role of ATP-dependent transporters. Nat Rev Cancer 2 (1) 48–58. 11902585 10.1038/nrc706

[24] Raaijmakers MH (2007) ATP-binding-cassette transporters in hematopoietic stem cells and their utility as therapeutical targets in acute and chronic myeloid leukemia. Leukemia 21: 2094–2102. 17657220 10.1038/sj.leu.2404859

[25] Wisser RJ, Kolkman JM, Patzoldt ME, Holland JB, Yu J, et al (2011) Multivariate analysis of maize disease resistances suggests a pleiotropic genetic basis and implicates a GST gene. Proc Natl Acad Sci 108 (18) 7339–7344. 21490302 10.1073/pnas.1011739108 PMC3088610

[26] Rudd LP, Kabler SL, Morrow CS, Townsend AJ (2011) Enhanced glutathione depletion, protein adduct formation, and cytotoxicity following exposure to 4-hydroxy-2-nonenal (HNE) in cells expressing human multidrug resistance protein-1 (MRP1) together with human glutathione *S*-transferase-M1 (GSTM1). Chemico-Biological Interactions 194 (2–3) 113–119. 21925487 10.1016/j.cbi.2011.08.012 PMC3221485

[27] Cohen JD, Tham KY, Mastrandrea NJ, Gallegos AC, Monks TJ, et al (2011) cAMP-dependent cytosolic mislocalization of p27(kip)-cyclin D1 during quinol-thioether-induced tuberous sclerosis renal cell carcinoma. Toxicol Sci 122 (2) 361–371. 21693435 10.1093/toxsci/kfr118 PMC3155088

[28] Ding K, Su Y, Pang L, Lu Q, Wang Z, et al (2009) Inhibition of apoptosis by downregulation of hBex1, a novel mechanism, contributes to the chemoresistance of Bcr/Abl+ leukemic cells. Carcinogenesis 30 (1) 35–42. 19028701 10.1093/carcin/bgn251

[29] Kater L, Claffey J, Hogan M, Jesse P, Kater B, et al (2012) The role of the intrinsic FAS pathway in Titanocene Y apoptosis: The mechanism of overcomingmultiple drug resistance in malignant leukemia cells. Toxicol In Vitro 26 (1) 119–124. 21986259 10.1016/j.tiv.2011.09.010

[30] Stege A, Priebsch A, Nieth C, Lage H (2004) Stable and complete overcoming of MDR1/P-glycoprotein-mediated multidrug resistance in human gastric carcinoma cells by RNA interference. Cancer Gene Ther 11 (11) 699–706. 15375376 10.1038/sj.cgt.7700751

[31] Walter RB, Gooley TA, van der Velden VH, Loken MR, van Dongen JJ, et al (2007) CD33 expression and P-glycoprotein-mediated drug efflux inversely correlate and predict clinical outcome in patients with acute myeloid leukemia treated with gemtuzumab ozogamicin monotherapy. Blood 109 (10) 4168–4170. 17227830 10.1182/blood-2006-09-047399 PMC1885511

[32] Fu D, Roufogalis BD (2007) Actin disruption inhibits endosomal traffic of P-glycoprotein-EGFP and resistance to daunorubicin accumulation. Am J Physiol Cell Physiol 292 (4) C1543–1552. 17122416 10.1152/ajpcell.00068.2006

[33] Goler-Baron V, Assaraf YG (2011) Structure and function of ABCG2-rich extracellular vesicles mediating multidrug resistance. PLoS One 6 (1) e16007. 21283667 10.1371/journal.pone.0016007 PMC3025911

[34] Lallemand D, Saint-Amaux AL, Giovannini M (2009) Tumor-suppression functions of merlin are independent of its role as an organizer of the actincytoskeleton in Schwann cells. J Cell Sci 122 (Pt 22) 4141–4149. 19910496 10.1242/jcs.045914

[35] Brown KL, Birkenhead D, Lai JC, Li L, Li R, et al (2005) Regulation of hyaluronan binding by F-actin and colocalization of CD44 and phosphorylated ezrin/radixin/moesin (ERM) proteins in myeloid cells. Exp Cell Res 303 (2) 400–414. 15652352 10.1016/j.yexcr.2004.10.002

[36] Molinari A, Calcabrini A, Meschini S, Stringaro A, Crateri P, et al (2002) Subcellular detection and localization of the drug transporter P-glycoprotein in cultured tumor cells. Curr Protein Pept Sci 3 (6) 653–670. 12470219 10.2174/1389203023380413

[37] Villar VH, Vögler O, Martínez-Serra J, Ramos R, Calabuig-Fariñas S, et al (2012) Nilotinib counteracts P-glycoprotein-mediated multidrug resistance and synergizes the antitumoral effect of doxorubicin in soft tissue sarcomas. Plos One 7 (5) e37735. 22662203 10.1371/journal.pone.0037735 PMC3360613

